# Cone Phosphodiesterase-6γ’ Subunit Augments Cone PDE6 Holoenzyme Assembly and Stability in a Mouse Model Lacking Both Rod and Cone PDE6 Catalytic Subunits

**DOI:** 10.3389/fnmol.2018.00233

**Published:** 2018-07-09

**Authors:** Wen-Tao Deng, Saravanan Kolandaivelu, Astra Dinculescu, Jie Li, Ping Zhu, Vince A. Chiodo, Visvanathan Ramamurthy, William W. Hauswirth

**Affiliations:** ^1^Department of Ophthalmology, University of Florida, Gainesville, FL, United States; ^2^Departments of Ophthalmology and Biochemistry, Center for Neuroscience, West Virginia University, Morgantown, WV, United States

**Keywords:** phosphodiesterase 6 (PDE6), rd10, Cpfl1, adeno-associated viral vector (AAV), achromatopsia, gene therapy, rod, cone

## Abstract

Rod and cone phosphodiesterase 6 (PDE6) are key effector enzymes of the vertebrate phototransduction pathway. Rod PDE6 consists of two catalytic subunits PDE6α and PDE6β and two identical inhibitory PDE6γ subunits, while cone PDE6 is composed of two identical PDE6α’ catalytic subunits and two identical cone-specific PDE6γ’ inhibitory subunits. Despite their prominent function in regulating cGMP levels and therefore rod and cone light response properties, it is not known how each subunit contributes to the functional differences between rods and cones. In this study, we generated an *rd10/cpfl1* mouse model lacking rod PDE6β and cone PDE6α’ subunits. Both rod and cone photoreceptor cells are degenerated with age and all PDE6 subunits degrade in *rd10/cpfl1* mice. We expressed cone PDE6α’ in both rods and cones of *rd10/cpfl1* mice by adeno-associated virus (AAV)-mediated delivery driven by the ubiquitous, constitutive small chicken β-actin promoter. We show that expression of PDE6α’ rescues rod function in *rd10/cpfl1* mice, and the restoration of rod light sensitivity is attained through restoration of endogenous rod PDE6γ and formation of a functional PDE6α’γ complex. However, improved photopic cone responses were achieved only after supplementation of both cone PDE6α’ and PDE6γ’ subunits but not by PDE6α’ treatment alone. We observed a two fold increase of PDE6α’ levels in the eyes injected with both PDE6α’ plus PDE6γ’ relative to eyes receiving PDE6α’ alone. Despite the presence of both PDE6γ’ and PDE6γ, the majority of PDE6α’ formed functional complexes with PDE6γ’, suggesting that PDE6α’ has a higher association affinity for PDE6γ’ than for PDE6γ. These results suggest that the presence of PDE6γ’ augments cone PDE6 assembly and enhances its stability. Our finding has important implication for gene therapy of PDE6α’-associated achromatopsia.

## Introduction

Cyclic nucleotide phosphodiesterases 6 (PDE6) belongs to the highly conserved mammalian PDEs composed of 11 different families that modulate cellular levels of the second messengers, cAMP and cGMP, by controlling their rates of degradation (Beavo, 1995; Francis et al., 2001). PDE6 is the key regulator of cytoplasmic cGMP concentration in rod and cone photoreceptor cells. Because of its primary role in controlling cGMP, regulation of PDE6 is essential for the speed, sensitivity, recovery and adaptation of visual signals (Burns and Arshavsky, 2005; Fu and Yau, 2007). Rod PDE6 is composed of two catalytic subunits α and β encoded by the *PDE6A*, *PDE6B* genes, and two inhibitory subunits γ encoded by *PDE6G*. In contrast, cone PDE6 is composed of two identical catalytic subunits of α’ encoded by *PDE6C* and two cone-specific inhibitory subunits γ’ encoded by *PDE6H* (Gillespie and Beavo, 1988; Hamilton and Hurley, 1990; Li et al., 1990).

Despite the important role it plays in photoreceptor function, the structure and function relationship of PDE6 is not well understood due to failure of its functional expression in various *in vitro* systems (Qin et al., 1992; Piriev et al., 1993, 2003; Qin and Baehr, 1994). Electron microscopic analysis of purified rod PDE6 catalytic dimers showed that the catalytic domains are located at the C-terminus, and are highly conserved among all known members of PDE family (Beavo, 1995). The N-terminal domains of PDE6 catalytic subunits consist of two regulatory cGMP binding GAF motifs (GAF A and GAF B) termed for their presence in cGMP-regulated PDE, adenylyl cyclases and the *E. coli* protein Fh1A (Aravind and Ponting, 1997). It is known that there is a synergistic effect between PDE6γ and cGMP binding to the catalytic subunits because: (1) PDE6γ binding to the catalytic subunits enhances the affinity of cGMP for the noncatalytic sites; and (2) when cGMP binds to the GAF domains, the affinity of PDE6γ for the catalytic subunits increases (Cote et al., 1994; Yamazaki et al., 2002). The region of PDE6γ responsible for this effect has been mapped to the central polycationic domain of PDE6γ known to have high affinity for the catalytic dimer (Artemyev and Hamm, 1992; Mou and Cote, 2001). Functional mapping of PDE6γ with the catalytic subunits showed that the C-terminus of PDE6γ is a key inhibitory domain. It interacts directly with the active site of PDE6 and blocks access of substrate to the catalytic pocket and therefore controls cGMP hydrolysis (Granovsky et al., 1997; Barren et al., 2009; Zhang and Artemyev, 2010). The middle region of PDE6γ stabilizes the overall binding affinity of PDE6γ with the catalytic subunits (Zhang et al., 2012).

One unique feature of rod PDE6 among the PDE family is that its catalytic subunits are composed of heterodimer αβ subunits. Evidence suggests that PDE6γ may form structurally and functionally distinctive interactions with the α- and β-subunits, and this asymmetric binding of PDE6γ to PDE6αβ may represent an important regulatory mechanism in rod phototransduction (Guo et al., 2005, 2006). The molecular mechanism underlying the distinctive photoresponses of rods vs. cones is poorly understood and is one of the fundamental questions remaining in photoreceptor biology. The potential roles of rod and cone PDE6 in phototransduction is of particular interest because PDE6’s functions in rods and cones as effector enzymes in controlling cGMP levels in the outer segments is key to fully understanding of rod and cone photoresponses. We have shown previously that rods of *rd10* mice expressing cone PDE6α’ delivered by an adeno-associated virus (AAV) vector are more sensitive to light than wild type rods, most likely due to the slower shutoff of their light responses (Deng et al., 2013). A similar study employing a transgenic approach showed that exchange of cone PDE6α’ for rod PDEαβ partially mimics the features of light adaptation (Majumder et al., 2015). Here, we study the association affinities among different subunits of PDE6 following AAV-mediated delivery in *rd10/cpfl1* mice. The *rd10/cpfl1* mice carry mutations in both rod PDE6β and cone PDE6α’, and they also lack rod PDE6αγ and cone PDE6γ’. We find that cone PDE6α’ has an intrinsically higher affinity for its partner PDE6γ’ than for PDE6γ, and the presence of PDE6γ’ augments cone PDE6 holoenzyme assembly and enhances its stability.

## Materials and Methods

### Animals

*Rd10, cpfl1*, and wild-type C57BL/6J mice were obtained from the Jackson Laboratory (Bar Harbor, ME, USA). *Rd10* and *cpfl1* mice were crossed to generate homozygous *rd10/cpfl1* mutant mice. Genotyping of *rd10* and *cpfl1* were performed according to previously published methods (Chang et al., 2007; Kolandaivelu et al., 2011). The mice were maintained in the University of Florida Health Science Center Animal Care Services Facilities in a continuously dark room, except for husbandry at ~400 lux illuminance. Physiological experiments were performed under dim red illumination using a Kodak number 1 Safelight filter (transmittance > 560 nm). All experiments were approved by the local Institutional Animal Care and Use Committees at the University of Florida and West Virginia University and conducted in accordance with the ARVO Statement for the Use of Animals in Ophthalmic and Vision Research and NIH regulations.

### Construction and Packaging of AAV Vectors

The murine PDE6γ’ cDNA was synthesized by GenScript (Piscataway, NJ, USA) and subcloned under the ubiquitous, constitutive small chicken β-actin promoter (smCBA; Haire et al., 2006) in self-complimentary AAV vectors to create sc-smCBA-mPDE6γ’. Construction of smCBA-mPDE6β and smCBA-mPDE6α’ were described previously (Deng et al., 2013). All constructs were packaged in AAV serotype 8-Y733F by transfection of H293 cells according to previously published methods (Zolotukhin et al., 1999).

### Subretinal Injections

All vectors were adjusted to the same titer of 5 × 10^12^ vector genomes/ml before injection. One microliter of vector was injected subretinally into the left eyes of *rd10/cpfl1* pups at postnatal 14 (P14) while the right eyes served as untreated controls. For co-injection, smCBA-mPDE6α’ and sc-smCBA-mPDE6γ’ vectors were mixed at a 2:1 ratio and 1 μl injected. Subretinal injections were performed as previously described (Pang et al., 2006, 2008). Briefly, eyes were dilated with 1% atropine (Akorn, Inc. Lake Forest, IL, USA) and 2.5% phenylephrine hydrochloride solution (Paragon Biotek, Portland, OR, USA). An aperture within the pupil area was made through the cornea with a 30 gauge disposable needle. A 33-gauge blunt needle mounted on a 5-μl Hamilton syringe (Hamilton Co., Reno, NV, USA) was then introduced through the corneal opening, avoiding the lens and reaching the subretinal space. Injections were visualized by fluorescein-positive subretinal bleb. 1% atropine eye drops and neomycin/polymyxin B/dexamethasone ophthalmic ointment (Bausch and Lomb Inc. Tampa, FL, USA) were given after injection.

### ERG Analysis

At 5 weeks post-injection, dark-adapted and light-adapted electroretinograms (ERGs) were recorded separately using a UTAS Visual Diagnostic System equipped with Big Shot Ganzfeld (LKC Technologies, Gaithersburg, MD, USA) according to protocols previously describe (Deng et al., 2013). Scotopic rod recordings were performed with three increasing light intensities at −1.6, −0.6, and 0.4 log cds/m^2^ after overnight dark adaption. Ten responses were recorded and averaged at each light intensity. Photopic cone recording were taken after mice were adapted to a white background light of 30 cds/m^2^ for 10 min. Recordings were performed with four flash intensities at 0.1, 0.7, 1.0 and 1.4 log cds/m^2^ in the presence of 30 cds/m^2^ background light. Fifty responses were recorded and averaged at each intensity. Scotopic and photopic b-wave amplitudes from untreated, treated *rd10/cpfl1* and wild-type controls at each intensity were averaged and used to generate a standard deviation.

### Immunohistochemistry and Morphology

Three days after ERG recordings, mice were sacrificed and eyes were enucleated for immunohistochemical analysis. Eyes were fixed in 4% paraformaldehyde at room temperature for 3 h. Cornea, lens and vitreous were removed from eyes without disturbing the retina. The remaining eyecup was rinsed with PBS and then cryoprotected by immersion in 30% sucrose in PBS for 4 h. Eyecups were then embedded in cryostat compound (Tissue TEK OCT, Sakura Finetek USA, Inc., Torrance, CA, USA) and frozen at −80°C. Embedded eyecups were sectioned at 12 μm thickness, rinsed in PBS, and blocked in 3% BSA, 0.3% Triton X-100 in PBS for 1 h at room temperature. Primary antibodies were: PDE6α’ (3184P, A polyclonal antibody raised in rabbit using purified His-tagged mouse PDE6α’ protein (amino acids 1–115) as the immunogen, gift of Dr. Visvanathan Ramamurthy, West Virginia University, Morgantown, WV, USA, 3184P denotes the rabbit number used for raising cone PDE6a’ antibody; Kirschman et al., 2010; Kolandaivelu et al., 2011), rhodopsin, blue-cone opsin and red/green-cone opsin (Millipore Bioscience Research Reagents). All primary antibodies were diluted 1:1000 in 1% BSA in PBS and incubated with sections overnight at 4°C. The sections were then washed three times with PBS, incubated with IgG secondary antibody tagged with Alexa-594 (Invitrogen) at 1:500 dilution and Lectin peanut agglutinin (PNA) conjugated to an Alexa Fluor 488 (Invitrogen) at 1: 200 dilution in PBS at room temperature for 1 h and washed with PBS. Sections were mounted with Vectashield Mounting Medium for Fluorescence (H-1000, Vector lab, In. Burlingame, CA, USA) and coverslipped. Sections were analyzed with a Zeiss CD25 microscope fitted with Axiovision Rel. 4.6 software.

For morphology analysis, eyecups were prepared as described above, then paraffin-embedded and sectioned at 4 μm through the optic nerve, followed by H&E staining.

### Immunoprecipitation (IP)

PDE6 complexes and total protein density were analyzed by immunoprecipitation (IP) with ROS-1 monoclonal antibody and immunoblotting. The frozen eyecups (3 each) were homogenized in 400 μl of IP buffer containing protease and phosphatase inhibitors and 10 mM iodoacetamide (10 mM Tris-HCl, pH 7.5, 100 mM KCl, 20 mM NaCl, 1 mM MgCl_2_) using a pellet pestle in a 1.5 ml Eppendorf tube on ice (5 s × 4). To solubilize the proteins, Triton X-100 was added to a final concentration of 1% and samples were incubated at 4°C for 30 min. Supernatants were collected by centrifuging at 10,000× *g* for 5 min at 4°C and incubated with 1.5 μg ROS-1 monoclonal antibody coupled protein A/G beads for 3 h. Unbound proteins were removed and beads were washed in wash buffer (1X PBS containing 0.1% Triton X-100). PDE6 subunits bound to ROS-1 were eluted from the beads by adding 1X SDS/PAGE sample buffer (62.5 mM Tris, pH 6.8, 2% SDS, 10% glycerol, 0.005% bromophenol blue, 5% 2-mercaptoethanol) and boiling for 5 min. Eluted proteins were separated by 4%–20% SDS-polyacrylamide gel (Bio-Rad) and immunoblotting was performed as described earlier with catalytic and inhibitory subunits specific rod or cone PDE6 antibodies (Kolandaivelu et al., 2011; Deng et al., 2013). RetGC1 antibody was obtained from Dr. David Garbers (Deceased, University of Southwestern Medical School, Dallas, TX, USA). Membrane scanning and protein densities were measured with an Odyssey Infrared Imaging System (LI-COR Biosciences). Images are representative of at least three independent experiments.

### Statistical Analysis

Scotopic a- and b-wave and photopic b-wave amplitudes at indicated flash intensities were compared by one-way ANOVA with the *post hoc* Bonferroni test to compare means at individual flash intensities. Protein quantifications were analyzed by one-way ANOVA with Bonferroni *post hoc* test to compare means of each treatment. Data were expressed as mean ± SEM.

## Results

### Histological Analysis of *rd10/cpf1* Mouse Retinas

**Figure 1:**
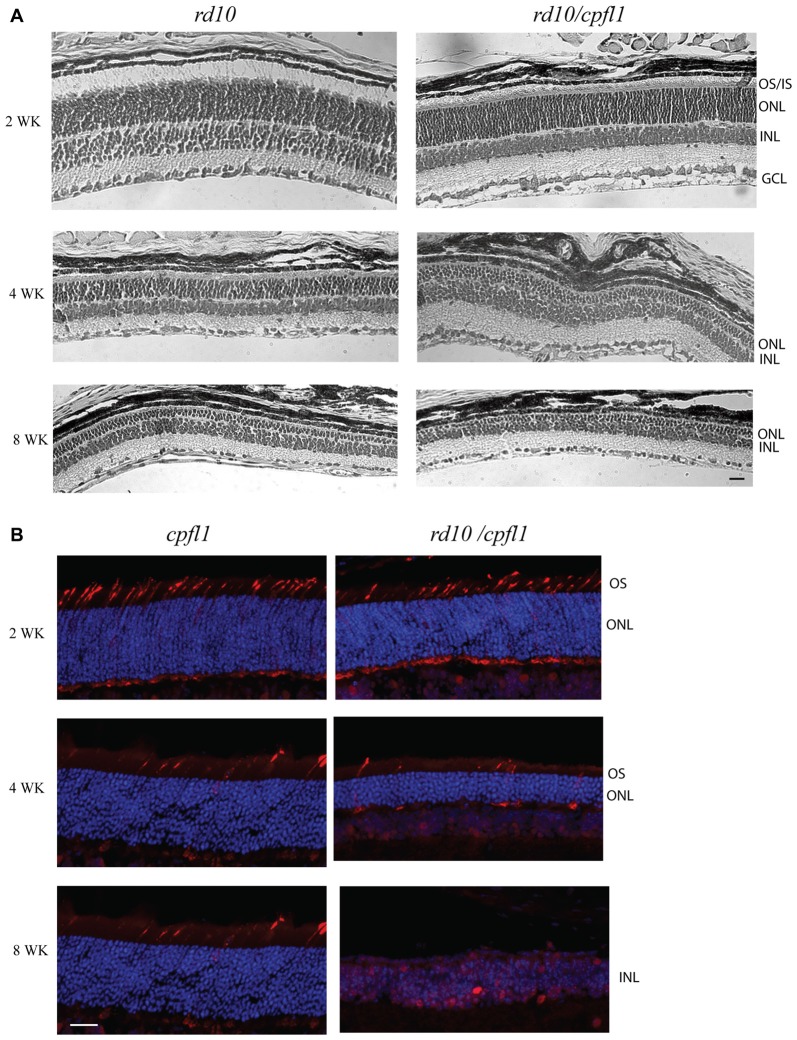
Histological analysis of *rd10/cpf1* mouse retinas. **(A)** Comparison of morphology of *rd10/cpfl1* to *rd10* by H&E staining. Rod photoreceptor cells degenerate with age as shown by thinning of ONL and loss of photoreceptor outer and inner segments. **(B)** Comparison of cone photoreceptor cell degeneration in *rd10/cpfl1* with *cpfl1* by anti-cone opsin antibody staining (labeled as red). OS/IS, outer/inner segments; ONL, outer nuclear layer; INL, inner nuclear layer; GCL, ganglion cell layer. Scale bar: 20 μm.

We first compared rod photoreceptor cell degeneration in *rd10/cpfl1* mice to *rd10* mice reared in the dark. Histological examination of hematoxylin and eosin (H&E) stained retinal sections revealed progressive outer nuclear layer (ONL) thinning in *rd10/cpfl1* retinas mirroring the retinal degeneration seen in *rd10* mice (Figure 1A). Dark-reared *rd10/cpfl1* mice showed similar ONL as in the wild-type mice 2 weeks of age, however, outer and inner segments appeared to be shorter. Retinal degeneration was rapid with only half of ONL remaining at 4 weeks. By 8 weeks, only 2–3 layers of ONL were present. We established that the rate of rod photoreceptor cell loss in *rd10/cpfl1* mouse is very similar to that of *rd10* mice, as previously reported (Chang et al., 2007).

We then characterized the rate of cone photoreceptor cell degeneration by comparing the cone opsin immunohistochemistry pattern in 2, 4 and 8 week-old *rd10/cpfl1* and *cpfl1* mice using a mixture of red/green- and blue-opsin antibodies. One previous study showed that although *cpfl1* retinas display grossly normal morphology and layering, there is vacuolization of a small subset of cells in the photoreceptor layer indicating cone loss as early as 3 weeks, with continued cone degeneration and complete cone loss by 10 weeks of age (Chang et al., 2009). *Rd10/cpfl1* mice show a cone opsin staining pattern similar to *cpfl1* at 2 weeks of age, followed by rapid cone photoreceptor cell degeneration with a majority of cones absent by 4 weeks of age. By 8 weeks, cone opsin staining was undetectable (Figure 1B). Cones of *rd10/cpfl1* mice degenerated slightly faster than those in *cpfl1* mice most likely due to concomitant rod degeneration. Furthermore, *rd10/cpfl1* mice exhibited both flat scotopic and photopic ERG responses at 3 weeks of age (data not shown).

### Rod and Cone Function in PDE6α’ and PDE6α’+PDE6γ’-treated *rd10/ cpfl1* Retinas

**Figure 2:**
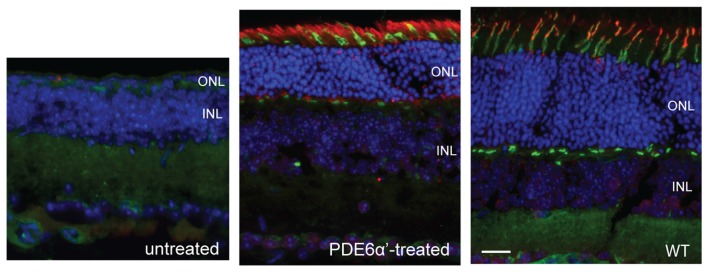
Detection of phosphodiesterase 6 (PDE6)α’ expression by immunofluorescence in *rd10/cpfl1* retinas following delivery of adeno-associated virus (AAV)-smCBA-PDE6α’. No PDE6α’ expression is detected in untreated eyes (left panel), while PDE6α’ (labeled as red) was expressed in both rods and cones in treated eyes (middle panel). PDE6α’ is expressed exclusively in cones in wild type mice (right panel). Cones were labeled by peanut agglutinin (PNA) as green. Note the much thicker ONL in the treated retina compared to that in the untreated control. Scale bar, 20 μm.

We first characterized the expression of cone PDE6α’ in *rd10/cpfl1* mice treated with AAV8 Y733F-smCBA-PDE6α’. The vector was subretinally delivered into one eye of postnatal 14 (P14) *rd10/cpfl1* mice, while the contralateral eyes remained untreated and served as controls. PDE6α’ expression was analyzed by immunostaining at 5 weeks post-injection. In C57BL/6 wild-type eyes, PDE6α’ is expressed specifically in cones (Figure 2). Untreated *rd10/cpfl1* eyes showed no PDE6α’ expression, whereas injected eyes showed robust PDE6α’ expression in both rods and cones.

**Figure 3:**
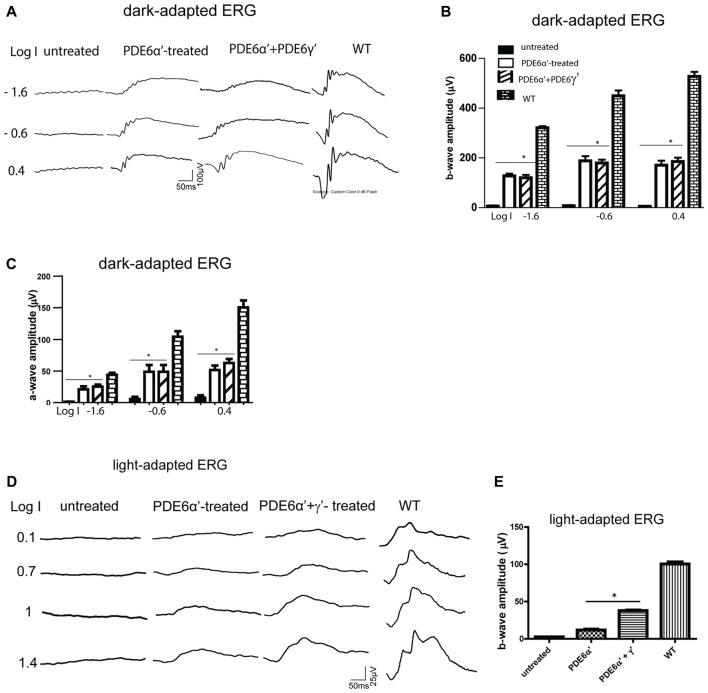
Scotopic and photopic electroretinogram (ERG) responses in *rd10/cpfl1* mice treated with PDE6α’ alone or PDE6α’ and PDE6γ’ together. **(A)** Representative examples of dark-adapted ERG traces from *rd10/cpfl1* mice at 5 weeks following delivery of AAV-smCBA-PDE6α’. **(B)** Dark-adapted b-wave is partially restored in injected *rd10/cpfl1* eyes. One-way ANOVA with the *post hoc* Bonferroni test demonstrated a significant difference between uninjected and contralateral vector-treated eyes at light intensities of −1.6, −0.6 and 0.4 log cds/m^2^ (data shown are mean ± SEM, *N* = 6, *p < 0.001 at all three light intensities, *F* = 253.0, 134.6 and 212.9 at −1.6, −0.6 and 0.4 log cds/m^2^, respectively). **(C)** Dark-adapted a-wave is partially restored in injected *rd10/cpfl1* eyes. One-way ANOVA with the *post hoc* Bonferroni test demonstrated a significant difference between uninjected and contralateral vector-treated eyes (mean ± SEM, *N* = 6, *p < 0.01 at all three light intensities, *F* = 28.34, 20.98 and 63.20 at −1.6, −0.6 and 0.4 log cds/m^2^, respectively). **(D)** Representative examples of full-field light-adapted ERG traces from *rd10/cpfl1* mice at 5 weeks following delivery of AAV-smCBA-PDE6α’ alone or co-injection of AAV-smCBA-PDE6α’ and scAAV-smCBA-PDE6γ’. **(E)** Photopic b-wave amplitudes are significantly higher in PDE6α’+γ’ co-injected eyes than eyes receiving PDE6α’ alone. The bar graph shows averaged ERG responses from 6 mice at a light intensity of 1.4 log cds/m^2^ (data shown are mean ± SEM, *N* = 6, *p < 0.01, *F* = 580.4).

We next characterized the rod function in PDE6α’- and PDE6α’+PDE6γ’-treated *rd10/cpfl1* eyes by full-field ERG analysis. Expression of PDE6α’ or PDE6α’+PDE6γ’ driven by smCBA promoter led to significant restoration of scotopic ERG responses at 5 weeks post-injection (Figures 3A–C). Both average b-wave and a-wave amplitudes in treated eyes are significantly higher than their undetectable ERG responses in contralateral untreated eyes at three light intensities tested. The average rod-mediated b-wave amplitude at a flash intensity of −1.6 log cds m^−2^ was 166 ± 14 μV (mean ± SEM) in PDE6α’-treated eyes, significantly higher than untreated controls (*n* = 6, *P* < 0.001, *F* = 253.0), and approximately 50% of those in the wild-type controls. The average rod-mediated a-wave amplitude at a flash intensity of −1.6 log cds m^−2^ was 21 ± 4.9 μV in PDE6α’-treated eyes, significantly higher than untreated controls (*n* = 6, *P* < 0.01, *F* = 28.34). Measured rod ERG responses were similar in eyes receiving PDE6α’ alone or PDE6α’ and PDE6γ’ mixture (*n* = 6, *P* > 0.05).

Rescue of rod photoreceptor cells in PDE6α’-treated eyes was also confirmed by examination of retinal morphology and immunostaining for rhodopsin. Hematoxylin and eosin staining (H&E) showed partial retinal structure preservation in treated eyes with 7–8 layers of photoreceptor nuclei retained in the ONL, while only one layer remained in contralateral untreated eyes. Retinal outer segments were also preserved in treated eyes (Supplementary Figure S1A). Consistent with this result, immunostaining with a rhodopsin antibody showed much stronger rhodopsin expression in treated eyes compared to almost undetectable staining in untreated eyes (Supplementary Figure S1B).

In contrast to robust rod rescue in *rd10/cpfl1* mice by PDEα’, rescue of cone function was significantly muted in PDE6α’-treated eyes (Figures 3D,E). To test the hypothesis that the presence of PDE6γ’ might assist proper folding of cone PDE6 holoenzyme and improve cone ERG rescue, PDE6γ’ driven by a smCBA promoter packaged in a fast-acting self-complimentary AAV vector was co-injected with PDE6α’. Improved photopic ERG responses were obtained with an average cone b-wave amplitude of 38 ± 1.5 μV (*n* = 6) at 1.4 log cds m^−2^, which was significantly higher than amplitudes from eyes treated with PDE6α’ alone (12 ± 1.9 μV, *P* < 0.001, *F* = 580.4). Immunostaining with M/S-opsin antibodies and PNA in co-injected eyes showed cone morphology rescue (Supplementary Figure S1C). Mild cone morphology rescue was also observed in PDE6α’-treated eyes (data not shown), however, these cones mostly existed because of slower degeneration due to rescue of rods. Our results suggest that the presence of PDE6γ’ from an earlier onset expression vector may help the proper folding, assembly, and stabilization of PDE6α’ more efficiently. The stability of the PDE6 α’γ’ complex could be vital for enhancing the survival and function of cones.

### Presence of PDE6γ’ Augments Cone PDE6 Assembly and Stability

**Figure 4:**
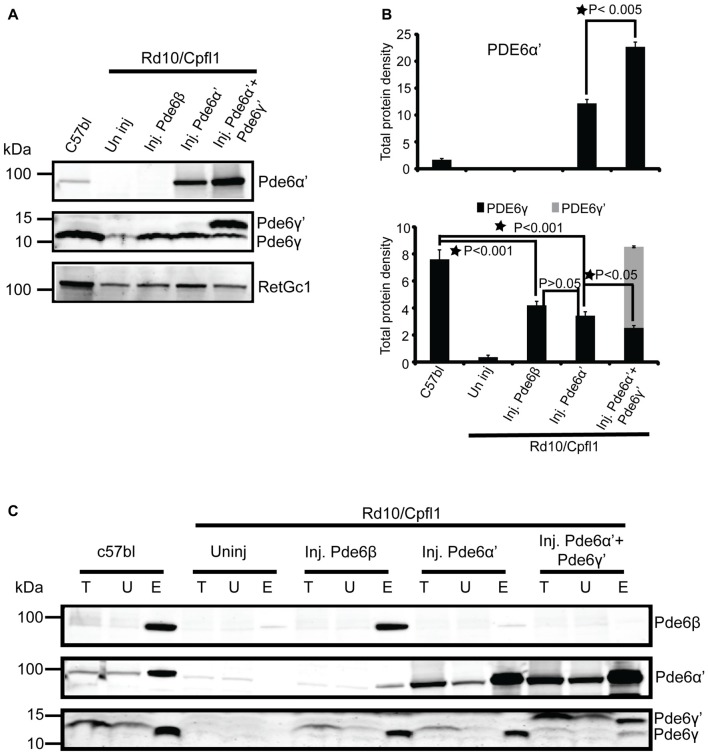
Cone PDE6 assembly and stability is enhanced when PDE6α’ and PDE6γ’ are co-expressed. **(A)** Immunoblotting of retinal extracts from wild type control, uninjected *rd10/cpfl1*, *rd10/cpfl1* mice injected with PDE6β, PDE6α’ and PDE6α’+PDE6γ’, and analyzed with the indicated antibodies. **(B)** Quantification of cone PDE6α’, rod PDE6γ and cone PDE6γ’ levels in retinal extracts from samples in **(A)**. Band densities were measured with a Li-COR Odyssey image analyzer. Statistical analysis show that PDE6α’ is significantly higher in PDE6α’+PDE6γ’-treated eyes than eyes treated with PDE6α’ alone (top panel, mean ± SEM, *N* = 3, *p < 0.005), whereas PDE6γ is reduced in PDE6α’+PDE6γ’-treated compared to eyes treated with PDE6α’ alone (bottom panel, mean ± SEM, *N* = 3, *p < 0.05, *F* = 53.44). There is no significant difference of PDE6γ levels between PDE6β- and PDE6α’-treated eyes (*N* = 3, *p* > 0.05). **(C)** Immunoblots show the assembled rod PDE6 and cone PDE6 when probed with ROS mouse monoclonal antibody from retinal extracts of wild type controls, uninjected *rd10/cpfl1*, and *rd10/cpfl1* mice injected with PDE6β, PDE6α’, and PDE6α’+PDE6γ’. Immunoblots were probed with indicated antibodies. Immunoprecipitation (IP) samples are 10 times more concentrated than input fractions. Protein molecular weight markers are shown on the left. T, Total; U, Unbound and E, Elution.

*Rd10/cpfl1* mice carry mutations in both rod PDE6β and cone PDE6α’. Next we investigated the levels of PDE6α’, PDE6γ’ and PDE6γ subunits by Western blot analysis from retinal extracts of C57Bl/6 wild-type control, uninjected *rd10/cpfl1*, and *rd10/cpfl1* injected with PDE6β, PDE6α’, or PDE6α’+PDE6γ’. We took advantage of the fact that cone specific PDE6γ’ has a slightly higher molecular weight than rod PDE6γ (Gillespie and Beavo, 1988) and used the same antibody to distinguish them. Western blot analysis showed that untreated *rd10/cpfl1* eyes lacked all three rod PDE6 subunits (Figure 4A; Supplementary Figure S2), and PDE6α’ treatment restored expression of rod PDE6γ subunits, similar to eyes treated with rod PDE6β (Figure 4A). However, cone PDE6γ’ expression was not detected in PDE6α’-treated eyes. In contrast, *rd10/cpfl1* eyes co-injected with PDE6α’+PDE6γ’ showed significantly higher levels of expression of both PDE6α’ and PDE6γ’ (Figure 4A). Qualitative analysis of the amount of PDE6α’, PDE6γ and PDE6γ’ from these samples showed that PDE6α’ levels were approximately double in co-injected eyes compared to eyes receiving only PDE6α’ (Figure 4B, top panel, *N* = 3, *P* < 0.005). The levels of PDE6γ in PDEβ- and PDE6α’-treated eyes were similar (*N* = 3, *P* > 0.05) and were about half of that in the wild-type control (*N* = 3, *P* < 0.001). However, the levels of PDE6γ were reduced (*N* = 3, *P* < 0.05) while the amount of PDE6γ’ was about twice as much as PDE6γ in co-injected eyes (Figure 4B, bottom panel). PDE6γ’ is delivered in half of the vector genomes of PDE6α’ (vector genome ratio 1:2). PDE6γ’ expression is expected to occur in both rods and cones following AAV vector delivery because we are using the nonspecific, ubiquitous smCBA promoter. However, we do not have a PDE6γ’-specific antibody to demonstrate this by immunohistochemistry. The higher PDE6α’ levels in co-injected eyes is mostly likely due to the presence of PDE6γ’.

Finally, we investigated the assembly status of PDE6 from the above samples by IP using ROS-I, a mouse monoclonal antibody that exclusively recognizes assembled and functional PDE6 complexes in both rods and cones (Kolandaivelu et al., 2009, 2011). We have shown previously that restoration of light sensitivity by cone PDE6α’ in rods of *rd10* mice was attributed to functional complex formation between cone PDE6α’ and rod PDE6γ (Deng et al., 2013). Here we confirm that rescue of rod function by PDE6α’ in *rd10/cpfl1* mice is also due to stabilization of endogenous PDE6γ and the resultant functional complex formation of PDE6α’γ (Figure 4C, Supplementary Figure S2). Interestingly, in PDE6α’+PDE6γ’ co-injected eyes, the majority of PDE6α’ coupled with PDE6γ’, with only a small amount of PDE6α’ associated with rod PDE6γ (Figure 4C), although PDE6γ was restored due to presence of PDE6α’. These results suggest that the reduced level of PDE6γ in co-injected eyes might be the result of degradation due to its reduced coupling to PDE6α’. Overall, these results clearly demonstrate that cone PDE6γ’ delivered with PDE6α’ specifically enhances cone PDE6 assembly and its stability, while assembly of endogenous rod PDE6γ with PDE6α’ is severely reduced in presence of PDE6γ’.

## Discussion

We studied the association among PDE6 subunits *in vivo* using *rd10/cpfl1* mice. The *rd10/cpfl1* mice have no detectable rod and cone ERG function, and carry mutations in both rod PDE6β and cone PDE6α’. Interestingly, these mice also lack rod PDE6αγ and cone PDE6γ’.The loss of both rod and cone PDE6 in these mice lead to early onset and rapid rod and cone photoreceptor cell degeneration. We show here that AAV vector-mediated expression of cone PDE6α’ driven by a constitutive promoter can rescue rod-mediated light responses and this functional rescue is achieved through stabilization of endogenous rod PDE6γ and complex formation of PDE6α’γ. However, improved photopic ERG responses were attained only in eyes co-injected with vectors expressing both PDE6α’ and PDE6γ’, but not in the eyes injected with the PDE6α’ vector alone. The levels of PDE6α’ are significantly higher in co-injected eyes and the majority of PDE6α’ complexed with PDE6γ’, although PDE6γ is also detected. These results demonstrate that PDE6α’ has higher intrinsic affinity for PDE6γ’ and its presence facilitates cone holoenzyme assembly and augmented its stability.

One of the biggest challenges hampering PDE6 research is failure of functional expression of this enzyme in various *in vitro* systems. It was recently shown that AIPL1 is an obligate chaperone required for heterologous expression of active PDE6α’ in cultured cells, and that the presence of PDE6γ dramatically increases the proportion of correctly folded, functional PDE6 produced in the presence of AIPL1 (Gopalakrishna et al., 2016). Our results showing that the presence of PDE6γ’ helps to stabilize cone PDE6 holoenzyme is consistent with this observation. In our experiment, we also find that PDE6γ’ increases the stability of PDE6α’ and facilitates cone PDE6 assembly demonstrated by the significant elevated amount of PDE6α’ as well as assembled and functional cone PDE6. We also found that levels of PDE6γ is reduced in co-injected eyes compared to eyes received PDE6α’ alone. We speculate that this reduction is the result of degradation due to its reduced coupling to PDE6α’ when PDE6γ’ is present. This is supported by previous studies suggesting that stability of PDE6 complex is dependent on the association of its interacting partners. First, in *rd10* mice carrying a mutation of PDE6β, PDE6α and PDE6γ were not detected (Deng et al., 2013). Second, AIPL1 interacts with PDE6α and the interaction is essential for PDE6 assembly. In the absence of AIPL1, all PDE6 subunits are degraded regardless of their normal synthesis (Kolandaivelu et al., 2009). Therefore, our data support the same conclusion that PDE6γ’ potentiates the proper folding of PDE6α’ and plays a critical role in the maturation of cone PDE6 holoenzyme.

An interesting question raised by this study is why PDE6γ’ enhances cone PDE6 assembly and stability compared with rod PDE6γ? A previous study demonstrated that the rod PDE6γ naturally exists in mouse cones and can couple with cone PDE6α’ to protect cone function and stability (Brennenstuhl et al., 2015). Our data show that the PDE6α’ can couple with PDE6γ when PDE6γ’ is absent. However, when PDE6γ’ is present along with the endogenous PDE6γ, PDE6α’ has a higher association affinity for its natural partner. Such a higher affinity might play a role in the faster light response kinetics of cone photoreceptor cells. Although the overall three-dimensional structure of the rod PDE6 holoenzyme was defined by using negative-stain electron microscopy, the quaternary structure of PDE6αβ as well as the interface between PDE6αβ and their inhibitory subunits PDE6γ remains elusive (Kameni Tcheudji et al., 2001; Goc et al., 2010). In addition, the heterogeneity of the rod PDE6α and β catalytic subunits in terms of their interactions with PDE6γ is poorly understood. There is evidence that PDE6γ may form distinctive structural and functional interactions with the PDE6α and PDEβ subunits (Guo et al., 2005). Despite the fact that rod and cone PDE6 subunits share similar domain organizations, the detailed structural relationships between PDE6α’ and γ’ is unknown and cone PDE6 may have a different mode of regulation and therefore display different enzymatic kinetics. Single cell recordings from rods expressing PDE6α’γ and PDE6α’γ’ would give detailed information regarding light signaling properties between the two types of rods and might reveal if PDE6γ’ plays a role in the faster light response of cones. The ideal model to perform this experiment would be a knock-in mouse line with endogenous PDE6γ replaced with cone PDE6γ’. In these mice, PDE6γ’ is expressed at the same level as PDE6γ in wild type mice, therefore detailed light response kinetics can be compared. Single cell recordings were not performed here due to the stochastic nature of AAV vector expression.

In our previous study, we showed that rod visual performance measured by optomotor responses was significantly improved in *rd10* mice treated with PDE6α’ but remained subpar compared to wild-type controls (Deng et al., 2013). We would expect similar improvement of scotopic visual acuity in *rd10/cpfl1* mice treated with PDE6α’ alone or a PDE6α’γ’ mixture. We also note that although improved photopic ERG responses were obtained with co-injection of vectors expressing both PDE6α’ and PDE6γ’, there is a possibility that these photopic recordings were partially derived from rods expressing cone PDE6α’γ’. These rods might be desensitized and could not be saturated by standard scotopic conditions, although our photopic ERG recordings were performed after longer than standard light exposure time for saturating normal rods. One approach to address this issue would be to express PDE6γ’ under a cone-specific promoter instead of a constitutive promoter such as CBA. However, our best cone-preferred promoter still confers some level of expression in the mouse rods (Dyka et al., 2014).

In summary, we show that cone PDE6α’ has an intrinsically higher affinity for its partner PDE6γ’ than for rod PDE6γ and that the presence of PDE6γ’ helps to stabilize cone PDE6 holo-enzyme and augment its assembly. This finding might be important for designing future gene therapy studies for treating patients with PDE6α’-associated achromatopsia.

## Author Contributions

W-TD and SK designed and performed the experiments, and wrote the manuscript. JL, PZ and VC conducted experiments. AD contributed to the data analysis. VR and WH contributed to study supervision. W-TD, SK, AD, VR and WH contributed to the critical revision of the manuscript.

## Conflict of Interest Statement

WH and the University of Florida have a financial interest in the use of AAV therapies, and WH owns equity in a company (AGTC Inc.) that might, in the future, commercialize some aspects of this work. The remaining authors declare that the research was conducted in the absence of any commercial or financial relationships that could be construed as a potential conflict of interest.

## Supplementary Material

The Supplementary Material for this article can be found online at: https://www.frontiersin.org/articles/10.3389/fnmol.2018. 00233/full#supplementary-material
