# Supplementary material for: Cone Phosphodiesterase-6γ’ Subunit Augments Cone PDE6 Holoenzyme Assembly and Stability in a Mouse Model Lacking Both Rod and Cone PDE6 Catalytic Subunits
Source: Front Mol Neurosci. 2018 Jul 9;11:233. doi: 10.3389/fnmol.2018.00233 (PMC6046437; doi:10.3389/fnmol.2018.00233)
Supplement: Supplementary file 1 [file Image_1.PDF]

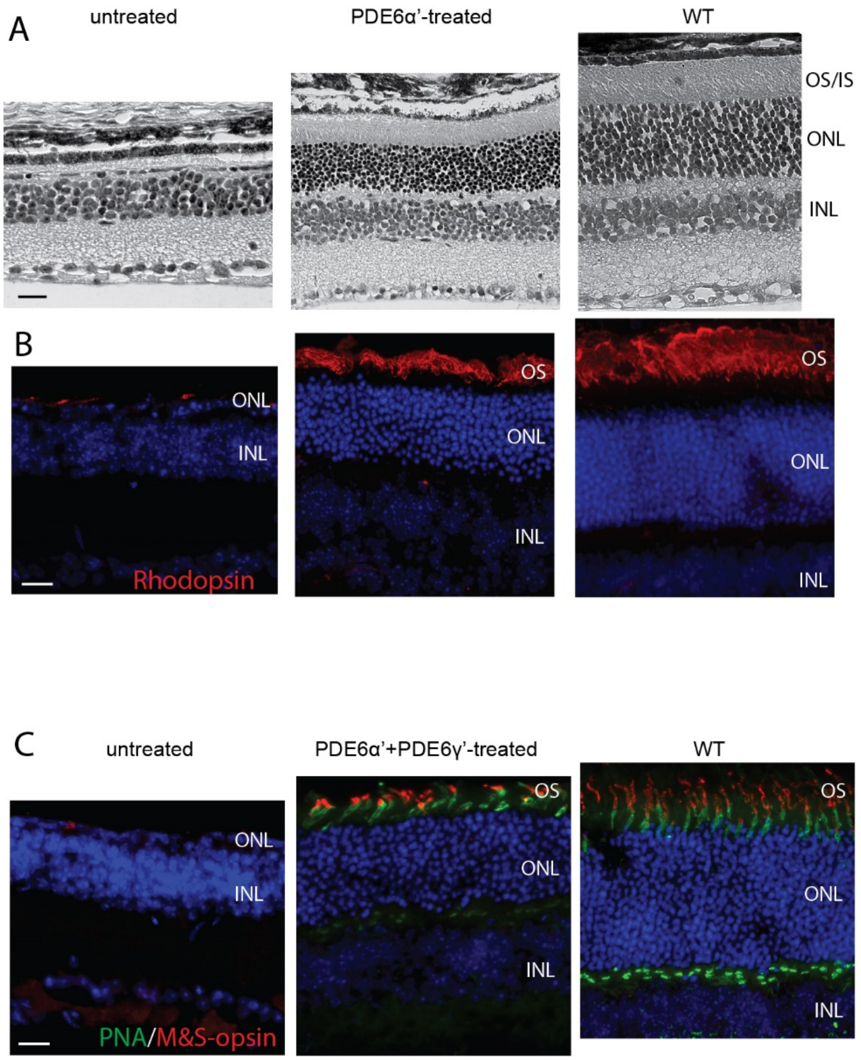

Figure S1. Retinal morphology is rescued following delivery of AAV-smCBA-PDE6α'. (A) Retinal morphology shows rescue of rod photoreceptor cells indicated by much thicker outer nuclear layer (ONL) and outer/inner segments (OS/IS) in treated eyes. (B) Immunohistochemistry by anti-rhodopsin antibody confirms the more abundant rhodopsin expression in treated *rd10/cpfl1* eyes relative to untreated controls. (C) Immunohistochemistry by anti-M/S-cone opsin antibodies demonstrates cone rescue in treated *rd10/cpfl1* eyes. Scale bar: 20 μm.

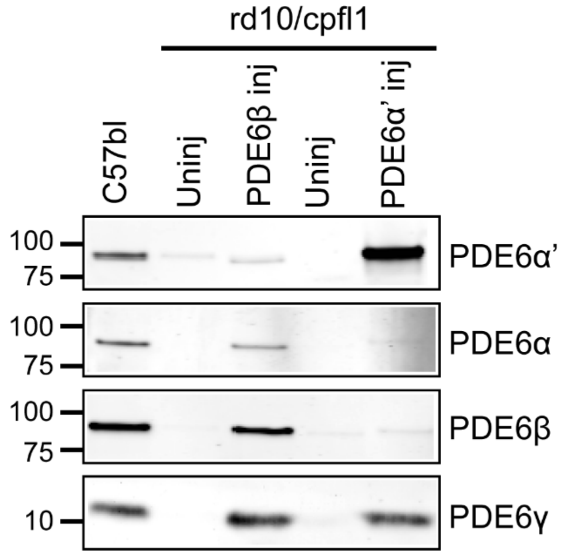

Figure S2. Injected rod PDE6β or cone PDE6α' in *rd10/cpfl1* retinas are assembled with endogenous rod PDE6γ subunit. Immunoblot analysis of assembled PDE6 using ROS1-IP from retina of wild type controls, uninjected *rd10/cpfl1*, and *rd10/cpfl1* mice injected with rod PDE6β or cone PDE6α'. The immunoblots were probed with indicated antibodies. A protein molecular weight scale is shown on the left.
